# Targeting the Gut–Mammary Axis for Understanding Mastitis Pathogenesis and Therapeutic Strategies

**DOI:** 10.3390/vetsci12111049

**Published:** 2025-11-01

**Authors:** Yan Li, Menghan Wang, Wenqiang Liu, Mingyang Geng, Mohammed Asiri, Fuad M. Alzahrani, Khalid J. Alzahrani, Qingshan Ma, Changfa Wang, Muhammad Zahoor Khan

**Affiliations:** 1 College of Agriculture and Biology, Liaocheng University, Liaocheng 252000, China; 2 Yili Kazak Autonomous Prefecture Livestock General Station, Yili 835000, China; 3 Department of Clinical Laboratory Sciences, College of Applied Medical Sciences, King Khalid University, Abha 61421, Saudi Arabia; 4 Department of Clinical Laboratories Sciences, College of Applied Medical Sciences, Taif University, Taif 21944, Saudi Arabia

**Keywords:** gut–mammary axis, microbiome, dairy cattle, dysbiosis, udder health, inflammation

## Abstract

Mastitis represents one of the most economically devastating diseases in dairy production, causing billions of dollars in annual losses through reduced milk quality and quantity. Recent advances in microbiome research have unveiled a critical gut–mammary axis that fundamentally influences mastitis susceptibility and pathogenesis in dairy cattle. Through comprehensive analysis of microbial communities across multiple anatomical sites, we demonstrate that mastitis development involves systematic disruption of both mammary and gastrointestinal microbiomes, characterized by reduced beneficial bacterial populations and increased pathogenic species. Healthy animals maintain balanced microbial ecosystems dominated by protective taxa including *Firmicutes*, *Bacteroidetes*, and beneficial *Lactobacillus* species, while mastitis-affected animals exhibit dysbiotic shifts toward *Proteobacteria* dominance, elevated *Streptococcus* and *Staphylococcus* populations, and compromised microbial diversity. Mechanistic investigations reveal that gut microbiota disruption compromises systemic immune competence, alters metabolite production including short-chain fatty acids and bile acids, and influences inflammatory mediators that circulate to mammary tissue. Therapeutic interventions targeting this axis, including probiotics, prebiotics, and plant-derived compounds, demonstrate significant efficacy in restoring microbiome homeostasis and reducing mastitis severity. These findings establish the gut–mammary axis as a fundamental regulatory mechanism in mastitis pathogenesis, opening new avenues for microbiome-based prevention and treatment strategies that could significantly enhance dairy health management while addressing antimicrobial resistance concerns.

## Simple Summary

This review explores the gut–mammary axis in bovine mastitis pathogenesis and management. Mastitis development involves systematic microbiome disruption characterized by altered Firmicutes-to-Proteobacteria ratios, depletion of beneficial bacteria (*Lactobacillus*, *Bifidobacterium*, *Akkermansia*), and pathogenic expansion of Staphylococcus and Streptococcus species. Intestinal dysbiosis promotes mastitis through compromised barrier integrity, reduced protective metabolite production (short-chain fatty acids, secondary bile acids), and inflammatory pathway activation. Fecal microbiota transplantation experiments definitively established causality by transferring dysbiotic gut communities from mastitis-affected animals to healthy recipients, successfully inducing mammary inflammation. These findings provide direct evidence of a causal relationship between gut microbiome disturbances and the development of mastitis. Therapeutic interventions including probiotics, prebiotics, polysaccharides, and plant-derived compounds restore microbiome homeostasis and reduce mastitis severity, offering sustainable alternatives to antibiotics for dairy production.

## 1. Introduction

Mastitis, characterized by inflammation of the mammary gland, represents one of the most economically significant and clinically challenging diseases affecting dairy cattle worldwide [1,2]. This multifactorial condition is primarily caused by bacterial pathogens that invade the udder tissue through the teat canal, leading to a cascade of inflammatory responses that compromise both milk quality and quantity [3]. Clinical manifestations of mastitis range from subclinical cases with no visible signs but elevated somatic cell counts and reduced milk production, to acute clinical presentations characterized by swelling, heat, pain, and redness of the affected mammary quarters, often accompanied by abnormal milk appearance including clots, flakes, or discoloration [4,5,6,7].

The economic burden imposed by this disease on the global dairy industry is staggering, with annual losses estimated between US$19.7 billion and US$32 billion, stemming from decreased milk yield, discarded milk, increased veterinary costs, premature culling, and reduced reproductive performance [8,9,10,11,12,13,14,15]. The United States alone accounts for approximately US$2 billion in annual losses [9,15], while Canada’s dairy industry faces Can$400 million (US$318 million) in losses each year, and China experiences fiscal impacts ranging from 15 to 45 billion Chinese Yuan (US$2.1–6.3 billion) [14]. These substantial economic consequences underscore the critical importance of effective prevention and treatment strategies for sustainable dairy production systems. While traditionally viewed as a localized bacterial infection, emerging evidence reveals that mastitis pathogenesis involves complex interactions between disrupted microbial communities and host immune responses [16,17,18].

**Figure 1:**
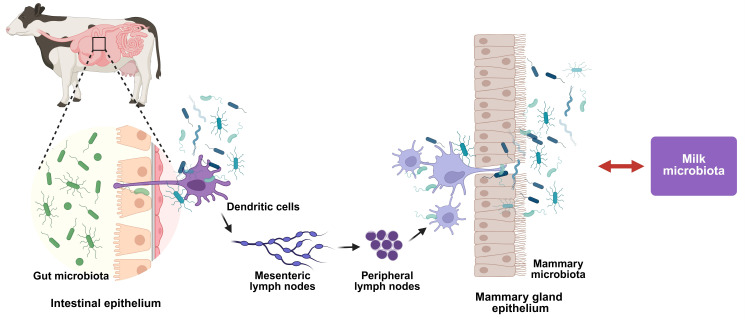
The enteromammary pathway: microbial translocation from gut to mammary gland. The figure presents the schematic representation of the enteromammary pathway showing how gut microorganisms can translocate across the intestinal epithelium, be captured by dendritic cells, migrate through lymph nodes, and ultimately colonize the mammary gland, contributing to milk microbiota composition.

This inflammatory condition can result not only from external microbial contamination but also from internal translocation of gut microbiota via the entero-mammary pathway (Figure 1) [16]. In this process, dendritic cells in the gut lamina propria capture microorganisms and transport them to mesenteric lymph nodes, where antigenic information is subsequently transmitted to mammary-associated lymph nodes through migrating lymphocytes, dendritic cell trafficking via lymphatic vessels, and soluble bacterial antigens in lymphatic fluid. Additionally, bacteria may translocate hematogenously through compromised intestinal barriers. These immune cell-mediated mechanisms ultimately facilitate microbial colonization of the mammary gland, contributing to mammary dysbiosis and milk microbiota composition. Recent microbiome research has fundamentally shifted our understanding of mastitis etiology, demonstrating that both mammary and intestinal microbial dysbiosis contribute to disease susceptibility [17,18]. Under normal physiological conditions, the mammary microbiota provides colonization resistance against pathogens through competitive exclusion and immune modulation, while the gut microbiome regulates systemic immune function and metabolic homeostasis [18,19,20,21]. However, environmental stressors, antibiotic exposure, and management practices can destabilize these microbial ecosystems, compromise natural defense mechanisms and create conditions favorable for pathogen establishment.

The gut–mammary axis, an emerging concept in veterinary and human medicine, suggests that the composition and metabolic activity of intestinal microbial communities can significantly influence mammary gland health and susceptibility to infectious diseases [22,23,24]. Gut dysbiosis can impair systemic immune competence and alter circulating metabolites that influence mammary gland susceptibility to infection. Conversely, mammary microbiota disruption directly undermines local immune responses, potentially leading to either excessive inflammation or inadequate pathogen clearance. Despite these advances, significant knowledge gaps persist regarding the mechanistic basis of gut–mammary communication, optimal microbiome restoration strategies, and the clinical translation of microbiome-targeted interventions [25,26,27]. Critical research priorities include identifying microbial biomarkers for early disease detection, characterizing temporal dynamics of microbiota changes during infection, and developing evidence-based therapeutic approaches that can be integrated into existing management protocols.

This review synthesizes current understanding of the gut–mammary axis and its role in mastitis pathogenesis, examining how bidirectional communication between intestinal and mammary microbiomes influences disease susceptibility and progression. We critically evaluate microbiome-mediated mechanisms underlying mastitis development, including disrupted colonization resistance, compromised barrier integrity, altered metabolite production, and dysregulated immune responses. Furthermore, we assess emerging microbiota-based therapeutic strategies—including probiotics, prebiotics, nutritional interventions, and plant-derived bioactive compounds—that target these mechanistic pathways to enhance udder health, reduce antimicrobial dependence, and promote sustainable dairy production systems. By integrating descriptive microbiome profiles with mechanistic insights into gut–mammary communication and immune modulation, this review provides a comprehensive framework for understanding mastitis pathogenesis and advancing precision microbiome-based interventions.

## 2. Comparative Microbiota of Milk and Gut in Healthy and Mastitis Animals

The contemporary understanding of mastitis pathogenesis has undergone a paradigmatic shift from the traditional conceptualization of a localized mammary gland infection to recognition of a complex multisystem disorder characterized by intricate microbiota–host interactions. This fundamental transformation in perspective has been driven by comprehensive investigations utilizing advanced sequencing technologies and metabolomic analyses, which have consistently demonstrated that mastitis development and progression are intimately linked to dynamic changes in microbial communities across multiple anatomical sites, thereby establishing the concept of a gut–mammary axis in dairy animal health.

### 2.1. Milk Microbiota Association with Mastitis

Across diverse dairy species and breeds, the most consistent hallmark of mastitis-associated dysbiosis is the dramatic alteration in *Firmicutes*-to-*Proteobacteria* ratios. However, the directional nature of this shift varies with pathogen type and disease severity. Multiple studies have documented this fundamental ecological disruption. For instance, Zhang L et al. [28] showed in yaks’ progression from balanced 39.7% *Firmicutes* and 60.17% *Proteobacteria* in healthy animals to 95.36% *Proteobacteria* dominance in clinical mastitis. In contrast, Salman et al. [29,30] found healthy cattle dominated by *Proteobacteria* (56.48%) but shifted to *Firmicutes* dominance (64%) during clinical mastitis. Furthermore, Secchi et al. [31] demonstrated pathogen-specific patterns. Specifically, *Streptococcus agalactiae* infections increased *Firmicutes* to 55.6% compared to 39.2% in controls. These findings, corroborated by Steinberg et al. [32] showing increased *Firmicutes* in subclinical mastitis across multiple breeds and Khasapane et al. [33] identifying Proteobacteria dominance in clinical cases, establish universal inflammatory-driven ecological shifts that transcend species boundaries while maintaining pathogen-specific characteristics.

The depletion of beneficial taxa represents another universal feature of mastitis dysbiosis, with multiple investigations identifying consistent losses of protective microorganisms across species. Healthy animals consistently harbor beneficial bacteria including *Leuconostoc mesenteroides*, *Lactococcus piscium*, *Carnobacterium maltaromaticum*, and *Lactococcus raffinolactis* in yaks [28], *Akkermansia muciniphila* and *Lactobacillus fermentum* in cattle [29,30], and protective taxa such as *Bifidobacterium pseudolongum*, *Jeotgalibaca porci*, and *Romboutsia* species [34]. Concurrently, studies by Tong et al. [35] and Burakova et al. [36] demonstrated that mastitis progression involves specific losses of *Proteobacteria*, *Actinobacteria*, *Acidobacteria*, as well as protective genera including *Ralstonia*, *Lachnospiraceae* NK3A20 group, *Acetitomaculum*, *Massilia*, and *Atopostipes*. The beneficial hub species identified by Derakhshani et al. [37], including *Bacteroidaceae* and *Phascolarctobacterium*, further emphasize the critical role of protective microbial networks in maintaining mammary health through anti-inflammatory metabolite production and immune homeostasis regulation.

Pathogenic microbial expansion follows predictable patterns across species, with certain taxa consistently emerging as mastitis-associated pathogens regardless of host species. *Staphylococcus aureus* appears as a dominant pathogen across studies, identified in yak mastitis [28], cattle investigations [38,39], and goat studies [40] where it maintained dominance across all disease states. *Streptococcus* species, including *S. agalactiae* [31,35] and *S. dysgalactiae* [29,30], consistently correlate with mastitis development, while opportunistic pathogens such as *Escherichia coli*, *Staphylococcus uberis* [39], *Moraxella osloensis*, *Psychrobacter cibarius* [28], and *Pseudomonas fluorescens* emerge with increasing disease severity. The pathogen-specific ecological signatures identified by Secchi et al. [31], where *Prototheca* spp. infections uniquely elevated *Cyanobacteria* to 17.3% versus 0.7% in controls, demonstrate that different etiological agents create distinct environmental niches favoring particular microbial communities.

Reduced microbial diversity emerges as a universal consequence of mastitis development, consistently documented across all species and study designs. Alpha diversity analysis by Salman et al. [29,30] showed reduced microbial diversity in mastitis cases across both Sahiwal cattle and Nili Ravi buffalo, while Zhang, L. et al. [28] documented progressive diversity loss correlating with yak mastitis severity. Maslennikova et al. [41] found mastitis-affected cows consistently harbored fewer bacterial taxa compared to healthy controls, with Polveiro et al. [40] demonstrating in goats that diversity disruption intensified with disease severity, reaching maximum alteration in gangrenous mastitis. This pattern is further supported by temporal studies showing that diversity reduction persists throughout lactation phases, suggesting that microbiome instability represents both a consequence and a perpetuating factor in mastitis pathogenesis.

The polymicrobial nature of mastitis has been consistently demonstrated across investigations, fundamentally challenging traditional single-pathogen therapeutic paradigms. Rudenko et al. [39] revealed that mastitis involves microbial consortia comprising two to seven distinct isolates with significant biofilm-forming capacity in 63.6% of cases, particularly among *Staphylococcus aureus*, *Escherichia coli*, and *Staphylococcus uberis*. This polymicrobial complexity is supported by Hoque et al. [42], who identified that mastitis-associated microbes carry genes for bacterial invasion, immune system disruption, antibiotic resistance, and oxidative stress induction, with 68% representing previously unreported opportunistic strains. The functional gene enrichment creates self-reinforcing inflammatory environments resistant to conventional therapies, while the reduced biofilm-forming capabilities of protective *Lactobacillus* species (22.5%) suggest competitive exclusion mechanisms that are disrupted during polymicrobial infections.

Specific diagnostic biomarkers have emerged from multiple studies, providing taxonomic signatures that could revolutionize mastitis detection and monitoring strategies. Complementary investigations identified overlapping biomarker panels including *Hymenobacter* and *Lachnospiraceae* NK4A136 group as mastitis indicators, increased *Bifidobacterium* and *Lachnospiraceae*_AC2044_group correlating with somatic cell counts of 100,000 cells/mL, and *Romboutsia*, *Turicibacter*, and *Paeniclostridium* as predictive markers for inflammatory development when somatic cell counts exceed 2 × 10^5^ [24,36,43]. These quantitative thresholds, combined with phylum-level shifts from beneficial *Actinobacteriota* to inflammation-associated *Firmicutes* and *Proteobacteria* dominance, provide integrated diagnostic approaches combining traditional inflammatory markers with microbial profiling.

Host genetic factors significantly influence microbiome composition and mastitis susceptibility, with multiple studies documenting breed-specific and genetic-dependent microbial patterns. However, mastitis has low heritability at approximately 0.16, indicating that while genetic factors play a role, environmental and management factors are more substantial contributors to mastitis development [44]. Tarrah et al. [45] demonstrated that Holstein cows with different estimated breeding values for feed efficiency and mastitis resilience harbored distinct communities, with *Aerococcus*, *Corynebacterium*, *Facklamia*, and *Psychrobacter* more abundant in high feed efficiency animals, while *Mycoplana* and *Rhodococcus* predominated in low mastitis resilience groups. These genomic–microbiome interactions, combined with species-specific baseline differences documented across cattle, buffalo, yak, and goat studies, suggest that effective mastitis prevention strategies must integrate universal microbiome principles with host-adapted therapeutic approaches.

The mechanistic validation linking specific taxa to inflammatory tissue destruction provides direct evidence for microbiome-mastitis causality. Chen et al. [46] established quantitative correlations between *Corynebacterium kroppenstedtii* abundance and breast abscess formation rates, while Sokolov et al. [38] identified pathogenic consortia including *Staphylococcus aureus*, *Aerococcus* spp., and *Streptococcus* spp. directly associated with elevated somatic cell counts. These findings, combined with the temporal dynamics documented by Maslennikova et al. [41] showing consistent taxonomic reduction throughout lactation phases, establish that specific microbial signatures both predict and mechanistically contribute to inflammatory mastitis development. The relationship between pathogenic microbial expansion and mastitis development is primarily causative rather than merely correlational. Bacterial challenge studies demonstrate that introducing specific pathogens (*S. aureus*, *E. coli*, *Streptococcus* species) into healthy mammary glands successfully induces mastitis and associated dysbiotic shifts, confirming that pathogenic bacteria drive disease development. Additionally, successful antimicrobial treatment restores balanced microbial communities [36], with pathogen elimination preceding microbiome normalization. While bidirectional effects between systemic inflammation and microbial composition are theoretically possible, experimental evidence—including challenge studies, antibiotic trials, and restoration interventions—supports a predominantly unidirectional causative pathway where pathogenic proliferation drives inflammatory dysbiosis and disease progression.

Collectively, these comprehensive investigations establish mastitis as a predictable ecosystem-level disruption characterized by consistent dysbiotic patterns across species: *Firmicutes-Proteobacteria* ratio alterations, beneficial taxa depletion (particularly *Lactobacillus*, *Leuconostoc*, *Lactococcus*, *Akkermansia*, and *Bifidobacterium* species), pathogenic expansion (dominated by *Staphylococcus aureus*, *Streptococcus* species, and opportunistic organisms), reduced microbial diversity, and polymicrobial biofilm formation. The identification of universal protective and pathogenic taxa, combined with species-specific variations and host genetic influences, provides a comprehensive foundation for developing precision microbiome-targeted interventions that restore protective networks while eliminating pathogenic consortia through integrated therapeutic approaches.

### 2.2. Gut Microbiota Association with Mastitis

The concept of a gut–mammary axis has gained substantial empirical support through multiple independent studies demonstrating that gastrointestinal microbial dysbiosis plays a crucial role in mastitis development. Yu J et al. [47] provided compelling evidence for this axis by demonstrating that cows with subclinical mastitis harbored distinctly altered gut microbial communities characterized by increased abundance of potentially harmful bacteria including *Cyanobacteria*, *Proteobacteria*, *Succinivibrio*, and *Lactobacillus iners*, while beneficial symbiotic bacteria such as *Paraprevotella*, *Coprococcus*, *Succiniclasticum*, *Desulfovibrio*, and *Bifidobacterium pseudolongum* were significantly reduced. This microbial imbalance extends beyond compositional changes to functional metabolic disruptions, as evidenced by elevated pro-inflammatory metabolites in both feces and plasma, including 9(S)-HPODE, 25-hydroxycholesterol, and histamine, while anti-inflammatory compounds such as mandelic acid, gamma-tocotrienol, and deoxycholic acid were notably decreased.

The rumen microbiota has emerged as a particularly critical component of this gut–mammary axis, with Zhang, H. et al. [48] demonstrating that cows with high somatic cell counts exhibited significantly compromised milk quality alongside altered rumen microbial communities characterized by increased abundances of *Bacteroidetes*, *Firmicutes*, *Lachnospiraceae*, *Prevotella*, and *Rumiclostridium*. These animals also showed elevated serum inflammatory markers and enrichment in metabolic pathways related to glutathione metabolism and cellular signaling, with specific bacterial families serving as key mediators linking rumen fermentation status, inflammatory responses, and milk quality parameters. This finding aligns with the work of Chuang et al. [49], who identified specific ruminal bacterial taxa as potential biomarkers for mastitis, with pathogenic genera such as *Sharpea* correlating positively with somatic cell counts and pro-inflammatory cytokines, while beneficial bacteria including *Ruminococcaceae* UCG-014, *Ruminococcus flavefaciens*, and *Treponema saccharophilum* demonstrated negative associations with inflammatory markers. Critical metabolic mediators have been identified that link gut dysbiosis to mastitis pathogenesis. Zhao, C. et al. [50] identified sialic acid as a crucial metabolic fuel that exacerbates gut dysbiosis and drives mastitis development, with cows experiencing subacute ruminal acidosis-associated mastitis exhibiting elevated sialic acid levels and distinct microbial communities characterized by increased pathogenic *Moraxellaceae* and reduced beneficial *Prevotellaceae*. Experimental validation demonstrated that sialic acid treatment specifically induced mastitis only when gut dysbiosis was present, associated with enhanced growth of harmful bacteria including *Escherichia coli*, *Enterobacteriaceae*, and *Akkermansiaceae*, while interventions targeting pathogenic *Enterobacteriaceae* or supplementing with beneficial *Lactobacillus reuteri* effectively alleviated mastitis symptoms. Furthermore, sialic acid potentiated serum LPS levels caused by antibiotic treatment, leading to increased activation of the TLR4-NF-κB/*NLRP3* pathways in the mammary gland and colon.

The translocation of pathogenic microorganisms from the gastrointestinal tract to the mammary gland represents a key mechanism underlying the gut–mammary axis. Hu et al. [51] demonstrated that subacute rumen acidosis increased permeability across multiple barriers including blood-milk, gut, and rumen barriers, allowing harmful substances to translocate between body compartments. Lipopolysaccharides from the disrupted gut microbiota entered the bloodstream and accumulated in mammary glands, triggering inflammatory responses that led to mastitis symptoms, with *Stenotrophomonas* bacteria identified as particularly important in this process. Oral administration of this organism to lactating mice successfully induced mastitis, confirming its pathogenic role in endogenous infection routes.

Neuronal–microbial interactions add another layer of complexity to gut–mammary axis regulation. He, Y. et al. [52] demonstrated that vagotomy induces blood–milk barrier disruption and mammary gland inflammation through gut microbiota-dependent mechanisms, with vagotomy significantly altering gut microbial composition by reducing beneficial bacteria (*Firmicutes*) while increasing pathogenic bacteria (*Proteobacteria*, *Campylobacterota*, and *Rikenellaceae_RC9_gut_group*). The study revealed that vagotomy disrupts tryptophan metabolism, reducing 5-hydroxyindole acetic acid levels, and that supplementation with this metabolite effectively alleviates mastitis through activation of the aryl hydrocarbon receptor (AhR) and subsequent inhibition of the NF-κB inflammatory pathway.

The severity of mastitis correlates directly with the degree of gut microbiota dysbiosis, as demonstrated by Wang, Y. et al. [53], who found that cows with clinical mastitis showed increased inflammation-associated bacteria including *Lachnospiraceae*, *Moraxella*, and *Neisseriaceae*, accompanied by elevated inflammatory metabolites. Subclinical mastitis exhibited increased opportunistic pathogens (*Ruminiclostridium_9* and *Enterorhabdus*) along with antibacterial compound-producing metabolites, while beneficial microbes were depleted across both mastitis groups, specifically short-chain fatty acid-producing bacteria (*Prevoterotoella_1*) and probiotics (*Bifidobacterium*). This experimental proof-of-concept is mechanistically supported by Hu et al. [54], who showed that gut microbiota dysbiosis significantly worsened *S. aureus*-induced mastitis severity through compromised blood–milk barrier function, with restoration achieved through short-chain fatty acid supplementation that modulated local inflammatory responses.

The cellular and molecular mechanisms underlying microbiota-mediated immune protection involve complex interactions between commensal bacteria and immune surveillance systems. Pan et al. [55] demonstrated that commensal microbiota-dependent γδ T cells (Tregs) mediate *IL-17A*-driven neutrophil recruitment essential for defense against *S. aureus* mastitis. Antibiotic treatment revealed that microbiota disruption caused significant downregulation of *IL-17A* signaling pathway-related genes, with the relative abundance of *Firmicutes* decreasing significantly while *Proteobacteria*, *Bacteroidota*, and *Campylobacterota* increased, establishing that beneficial microbes maintain protective inflammatory surveillance through the γδ T cells/*IL-17A* axis.

Systemic inflammatory cascades represent the downstream consequences of gut–mammary axis disruption. Guo et al. [56] demonstrated that mastitic cows exhibited significantly elevated concentrations of inflammatory markers including TNF-α, IL-1, and LPS compared to healthy animals, with these systemic inflammatory changes correlating directly with structural differences in rumen microbial communities including increased *Aeriscardovia*, *Lactococcus*, and *Bacillus* in healthy cow feces compared to mastitic animals. The identification of *Moryella* as a characteristic bacterial signature in mastitic animals suggests that specific microbial taxa may trigger or sustain inflammatory cascades that extend beyond the mammary gland. Chen et al. [57] further revealed that mastitis-associated gut microbiota dysbiosis, particularly increased *Proteobacteria* abundance, triggered cascading metabolic disruptions affecting bile acid metabolism, amino acid metabolism, and arachidonic acid pathways in mammary tissue, ultimately activating inflammatory proteins including *IFIH1*, *Tnfaip8l2*, *IRGM*, and *IRF5*.

The diversity–inflammation relationship represents a critical determinant of mastitis susceptibility, with reduced microbial diversity correlating with enhanced inflammatory activity. Zhu et al. [58] revealed that clinical mastitis cows showed significantly reduced fecal microbial diversity and disturbed milk microbiota compared to healthy controls, with metabolomic analysis identifying extensive disruptions in amino acid and energy metabolism pathways that fuel inflammatory processes. Clinical mastitis cows showed increased fecal abundance of UCG-010, *Bacteroides*, *Prevotella*, *Ruminococcus*, *Ralstonia*, and seven other genera, but decreased dgA-11 gut group, while their milk had elevated pathogenic *Proteobacteria* and reduced *Bilophila* and *Clostridium innocuum* group compared to healthy cows with higher beneficial *Firmicutes*, *Actinobacteriota*, *Bifidobacterium*, and *Streptococcus*.

High-throughput sequencing studies demonstrate that mastitis development involves paradoxical changes in microbial communities, characterized by decreased overall diversity coupled with increased species abundance, suggesting selective proliferation of specific bacterial populations rather than generalized microbial overgrowth [59]. The identification of significantly altered genera in both milk (*Sphingomonas*, *Stenotrophomonas*) and fecal samples (*Alistipes*, *Flavonifractor*, *Agathobacter*, *Pygmaiobacter*) provides strong evidence for an endogenous microbial pathway linking intestinal and mammary gland health. In buffalo, mastitis-associated microbiota changes manifest primarily through specific taxa alterations, with increased abundance of uncultured_bacterium_f_*Muribaculaceae* and *Eubacterium_nodatum_group*, alongside decreased beneficial genera including *Ruminococcus_2* and *Turicibacter* [60].

A critical mechanistic pathway linking gut health to mastitis susceptibility involves bile acid metabolism, as demonstrated in subacute ruminal acidosis (SARA) models. Zhao, C. et al. [61] showed that SARA cows exhibited significantly reduced levels of both primary bile acid cholic acid (CA) and secondary bile acid deoxycholic acid (DCA) in their milk compared to healthy cows, directly associated with increased susceptibility to *Staphylococcus aureus*-induced mastitis. The mechanistic pathway involves healthy gut microbiota converting CA to DCA, leading to TGR5 activation, cAMP-PKA signaling, inhibition of NF-κB/NLRP3 pathways, and ultimately reduced inflammation and mastitis protection. Vancomycin-induced gut dysbiosis recapitulated this phenotype by causing marked alterations in gut microbiota structure, functionally resulting in reduced DCA levels, impaired *TGR5* activation in mammary tissue, and more severe mastitis symptoms.

### 2.3. Fecal Microbiota Transplantation Causes Mastitis

The transmissible nature of mastitis-associated microbial communities has been conclusively demonstrated through fecal microbiota transplantation experiments, providing definitive proof that dysbiotic microbial communities possess inherent pathogenic properties that can directly cause disease across mammalian hosts. Hoque et al. [62] successfully induced mastitis in mice by transferring microbiota from clinical mastitis cows, with whole metagenome sequencing revealing significant differences in microbiome diversity and composition between clinical mastitis and healthy samples. The mastitis-associated microbiomes were characterized by specific bacterial species such as *Pseudomonas aeruginosa* and *Lactobacillus crispatus* in cows, while mastitis-induced mice showed enrichment of *Muribaculum* spp., *Escherichia coli*, and *Staphylococcus aureus* among others, establishing that microbiome dysbiosis and genomic functional perturbations serve as central mechanisms in mastitis pathogenesis. Despite the successful transmission of mastitis syndrome across species, the microbial specificity reveals important host-dependent variations in pathogenic community structure. Cows and mice shared only 1.14% of mastitis-associated microbial taxa, indicating species-specific microbiome patterns while maintaining conserved mastitis syndromes across mammalian hosts [62]. This finding suggests that while the functional consequences of dysbiosis are conserved, the specific bacterial actors may vary according to host physiology and environmental factors, pointing to universal mechanisms of microbiota-mediated disease pathogenesis rather than specific pathogen-driven processes.

The mechanistic pathways underlying microbiota transplantation-induced mastitis have been elucidated through detailed molecular analysis of recipient animals. Zhao C et al. [63] demonstrated that cows with clinical mastitis exhibited marked systemic inflammation associated with significant ruminal dysbiosis, particularly characterized by enriched *Proteobacteria* in the rumen. Critically, ruminal microbiota transplantation from mastitis cows to mice resulted in recipient mice developing mastitis symptoms along with increased mammary pro-inflammatory signature activation through TLR4-cGAS-STING-NF-κB/*NLRP3* pathways, establishing that the inflammatory cascade activation represents a conserved response to dysbiotic microbial signals regardless of the original microbiota source.

The protective role of beneficial commensal bacteria emerges as a crucial counterbalance to pathogenic microbiota effects. Zhao C et al. [23] demonstrated that cows with mastitis had marked gut dysbiosis characterized by enrichment of opportunistic pathogenic *Escherichia_Shigella* and depletion of commensal *Roseburia*. When fecal microbiota transplantation from donor cows with mastitis was performed in recipient mice, it significantly induced mastitis and facilitated the translocation of pathobionts from the gut to the mammary gland. However, supplementation with commensal *Roseburia intestinalis* improved mastitis and microbial dysbiosis by producing butyrate, which was associated with inflammatory signaling inhibition and barrier repair, effectively limiting bacterial translocation. This finding demonstrates that the balance between pathogenic and protective microbial populations determines the severity of transplantation-induced mastitis. The causal relationship between microbiota dysbiosis and inflammatory mastitis susceptibility extends beyond bovine models to human lactation mastitis. Kong et al. [64] provided definitive evidence that dysbiotic gut microbiota actively contribute to lactation mastitis by demonstrating that fecal microbiota transplantation from mastitis patients induced systemic inflammation in recipient mice, characterized by elevated pro-inflammatory cytokines IL-4, IL-17, MPO, IL-6, IL-1β, and TNF-α. The microbiota alterations in mastitis patients included an increased *Firmicutes/Bacteroidetes* ratio, indicating dysbiosis, with elevated *Actinobacteria* and reduced *Verrucomicrobia* at the phylum level. At the genus level, beneficial bacteria *Ruminococcus* and *Faecalibacterium* were significantly decreased, whereas *Parabacteroides* were increased in both mastitis patients and mice that received their fecal transplants, establishing conserved dysbiotic patterns across human and experimental animal models.

The relationship between metabolic dysfunction and mastitis susceptibility has been further clarified through microbiota transplantation studies linking subclinical ketosis to mammary gland inflammation. Tang R et al. [65] demonstrated that subclinical ketosis in dairy cows exhibits profound associations with elevated somatic cell counts in milk, accompanied by distinct alterations in rumen microbiota diversity and volatile fatty acid profiles. Innovative microbiota transplantation experiments provided compelling evidence that transferring rumen microbiota from subclinical ketosis and mastitis-affected cows into mice successfully induced mammary inflammation and liver function impairment, whereas transplants from healthy animals produced no such deleterious effects. Linear discriminant analysis identified key bacterial genera including *Chrysobacterium*, *Christensenellaceae_R-7_group*, *ceae_RC9_gut_group*, and *Prevotella* as significant biomarkers distinguishing the rumen microbiota of ketotic cows from healthy animals, indicating that metabolic disorders create specific dysbiotic signatures that predispose to mastitis development.

While the predominant evidence supports gut dysbiosis as a primary driver of mastitis susceptibility, potential bidirectional interactions cannot be entirely excluded. Systemic inflammation from mastitis could theoretically influence gut microbiota composition through altered immune signaling and metabolic changes. However, experimental evidence from fecal microbiota transplantation studies definitively demonstrates that gut dysbiosis alone is sufficient to induce mastitis in healthy recipients [62,63,64,65], establishing causality in the gut-to-mammary direction. Moreover, temporal sequence studies show that induced gut dysbiosis precedes mastitis development [50,61], and antibiotic-mediated microbiota depletion exacerbates mastitis severity [54], collectively indicating that gut microbiome disturbances represent a primary causative factor rather than merely a consequence of mammary inflammation.

**Figure 2:**
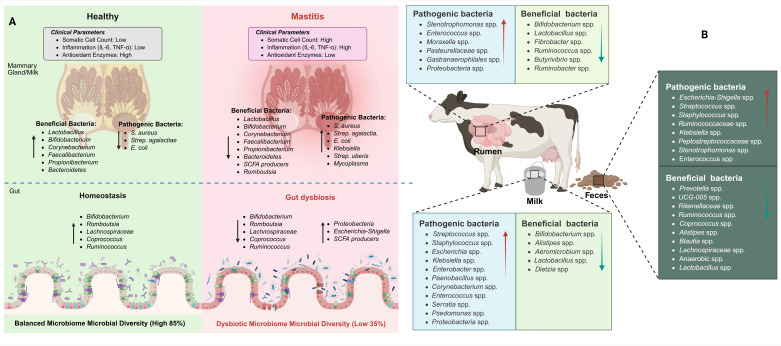
Bovine microbiome dysbiosis in mastitis: mammary gland pathology and systemic microbial distribution. (**A**): Comparison of mammary gland health status and associated microbiome profiles. Left panel shows healthy mammary tissue with low somatic cell count, reduced inflammation (IL-6, TNF-α), and high antioxidant enzyme activity, characterized by predominant beneficial bacteria (*Lactobacillus, Bifidobacterium, Faecalibacterium, Propionibacterium, Brevibacterium*) and maintained gut homeostasis with balanced microbiome diversity (85%). Right panel depicts mastitis condition with elevated somatic cell count, increased inflammation markers, decreased antioxidant enzymes, dominated by pathogenic bacteria (*S. aureus, Strep. agalactiae, E. coli, Klebsiella, Strep. uberis, Strep. dysgalactiae*), and associated gut dysbiosis with reduced microbial diversity (35%). (**B**): Anatomical distribution of beneficial and pathogenic bacteria across different bovine body sites. The diagram illustrates site-specific microbial communities in the rumen, mammary gland (milk), and intestinal tract (feces), showing the distinct bacterial populations that colonize each anatomical niche. Beneficial bacteria include *Bifidobacterium*, *Lactobacillus*, *Faecalibacter*, *Ruminococcus*, and *Butyrrivibrio* species, while pathogenic bacteria comprise *Staphylococcus*, *Enterococcus*, *Escherichia-Shigella*, *Streptococcus*, and other potentially harmful species specific to each body compartment.

While establishing this causal relationship, it is essential to contextualize the role of gut microbiota within the broader landscape of bovine health. It is important to note that gut microbiota dysbiosis has been implicated in numerous other disease conditions beyond mastitis, including metabolic disorders, lameness, respiratory infections, and reproductive diseases in dairy cattle. The gut microbiome serves as a central regulator of systemic health through modulation of immune function, metabolic homeostasis, and barrier integrity across multiple organ systems. Nevertheless, the specific mechanisms linking gut dysbiosis to mammary gland pathology—including bile acid metabolism disruption, bacterial translocation via dendritic cells, and alterations in circulating immunomodulatory metabolites—represent unique pathways that distinguish the gut–mammary axis from other microbiome–disease relationships. To provide a comprehensive overview of these relationships, a summary of microbiota associations between milk, gut, and fecal samples in relation to mastitis is provided in Table 1. In addition, Figure 2 presents the taxonomic classification of microbiota present in milk, fecal, and gut samples from both healthy and mastitis-affected animals.

### 2.4. Functional Correlation Between Gut and Milk Microbiota

The emerging concept of the gut–mammary axis posits that intestinal and mammary microbial communities are not isolated ecosystems but rather functionally interconnected through complex metabolic and immunological communication networks. This bidirectional crosstalk fundamentally influences mammary gland health and mastitis susceptibility, thereby establishing the gut microbiome as a critical determinant of udder immunity and disease resistance [17]. Central to understanding this axis is the recognition that the gut microbiome produces a diverse array of bioactive metabolites that enter systemic circulation and directly influence mammary gland physiology and immune function. Among these metabolites, short-chain fatty acids (SCFAs), particularly butyrate, propionate, and acetate, represent primary mediators of gut–mammary metabolic communication. These microbial fermentation products cross the intestinal epithelium, enter the bloodstream, and reach mammary tissue, where they exert multiple protective effects. Notably, butyrate has been shown to activate histone deacetylase 3 (HDAC3)-mediated antimicrobial programs in mammary macrophages, thereby enhancing pathogen clearance capacity [66]. Significantly, during mastitis, gut dysbiosis leads to reduced SCFA production, particularly butyrate depletion, which consequently compromises mammary antimicrobial defense mechanisms and creates permissive conditions for pathogen establishment.

In addition to SCFAs, secondary bile acids constitute another critical metabolic link between gut and mammary microbiomes. These compounds, produced through microbial transformation of primary bile acids in the intestinal lumen, modulate systemic inflammation through interactions with farnesoid X receptor (FXR) and TGR5, thereby influencing immune cell trafficking and cytokine production patterns relevant to mammary gland immunity [61]. Consistent with this mechanism, research has demonstrated that mastitis-associated gut dysbiosis alters bile acid metabolism profiles, reducing protective secondary bile acid production and contributing to systemic inflammatory status that predisposes animals to mammary infections [61].

Furthermore, tryptophan metabolism represents an additional metabolic axis connecting intestinal and mammary health. Gut microbiota converts dietary tryptophan into indole derivatives, including indole-3-propionic acid (IPA) and indole-3-acetic acid (IAA), which possess potent anti-inflammatory and barrier-protective properties [19,50]. These indole metabolites activate the AhR in immune cells, thereby promoting regulatory T cell differentiation and suppressing excessive inflammatory responses. Importantly, during mastitis development, alterations in gut microbial composition disrupt tryptophan metabolism, reducing protective indole metabolite production and compromising systemic immune regulation, which subsequently increases mammary inflammation susceptibility [50]. Building upon these findings, recent metabolomic investigations have identified succinate as a pro-inflammatory metabolite elevated during mastitis-associated gut dysbiosis. Specifically, Qiu et al. [67] demonstrated that gut microbiota-derived extracellular vesicles containing elevated succinate levels exacerbate mastitis severity in mice, thereby establishing a direct mechanistic link between intestinal metabolic dysregulation and mammary pathology. This finding exemplifies how altered gut microbial metabolism can actively promote mastitis development through systemic metabolite signaling.

Beyond these metabolic pathways, the gut–mammary axis operates through sophisticated immunological communication mechanisms that coordinate local and systemic immune responses. The intestinal immune system, housing approximately 70% of the body’s immune cells, serves as a primary training ground for immune competence that extends to peripheral tissues including the mammary gland [61]. Accordingly, gut microbiota composition critically influences the development, maturation, and functional capacity of immune cell populations that subsequently migrate to mammary tissue [21,50]. Within this immunological framework, dendritic cells (DCs) represent key cellular mediators of gut–mammary immunological communication [51,68]. These professional antigen-presenting cells sample intestinal microbial antigens, undergo maturation and activation, and migrate through lymphatic circulation to peripheral tissues including the mammary gland [69]. During this transit, DCs can carry viable commensal bacteria from the intestinal lumen to distant sites through a process termed bacterial translocation [70,71]. This enteromammary pathway allows beneficial gut microorganisms, particularly *Lactobacillus* and *Bifidobacterium* species, to colonize mammary tissue and contribute to the milk microbiome composition, providing natural protection against pathogens through competitive exclusion and immune modulation. However, it is important to note that gut dysbiosis can pervert this beneficial translocation mechanism, allowing pathogenic or opportunistic bacteria to access mammary tissue and contribute to mastitis pathogenesis [72]. Furthermore, they demonstrated that mastitis-associated changes in gut microbiota composition correlate with altered bacterial translocation patterns, potentially introducing dysbiotic microbial signatures into the mammary environment and compromising local immune homeostasis [72].

Complementing the role of dendritic cells, T lymphocyte populations represent another critical immunological link between gut and mammary compartments. Gut microbiota composition profoundly influences the balance between pro-inflammatory T helper 17 (Th17) cells and anti-inflammatory regulatory T cells. Specifically, a healthy, diverse gut microbiome promotes Treg development through mechanisms involving SCFA-mediated histone modifications and transforming growth factor-β (TGF-β) signaling, establishing systemic immune tolerance that prevents excessive inflammatory responses in peripheral tissues [73,74]. Conversely, dysbiotic gut microbiota skews differentiation toward Th17 cells, thereby promoting systemic inflammation and enhancing mammary tissue susceptibility to infection-induced damage. Moreover, immunoglobulin A (IgA), the predominant antibody isotype in mucosal secretions including milk, provides additional evidence of gut–mammary immunological connectivity [75]. The mammary gland receives IgA-producing plasma cells that originate from gut-associated lymphoid tissue (GALT) through a process termed the common mucosal immune system. These plasma cells, initially primed by intestinal microbial antigens, traffic to the mammary gland where they secrete IgA antibodies that recognize gut-derived microorganisms, thereby providing passive immunity to offspring while simultaneously contributing to mammary colonization resistance. Disruption of gut microbiome composition impairs optimal IgA responses, consequently compromising this critical protective mechanism [76].

Expanding our understanding of these gut–mammary communication mechanisms, recent research has identified microbiome-derived extracellular vesicles (mEVs) as novel mediators of this axis. These nanoscale lipid-bound particles, secreted by both commensal and pathogenic bacteria, contain diverse cargoes including proteins, lipopolysaccharides, nucleic acids, and metabolites that can exert biological effects on distant tissues [77]. In a seminal study, Qiu et al. [67] demonstrated that gut microbiota-derived EVs accumulate in mammary tissue during mastitis and contribute to disease pathology through delivery of pro-inflammatory molecules including succinate. Conversely, EVs derived from beneficial bacteria such as *Akkermansia muciniphila* have shown therapeutic potential in mastitis management, suggesting that mEVs represent both pathogenic mediators and potential therapeutic vehicles within the gut–mammary axis.

Critically, longitudinal studies employing fecal microbiota transplantation (FMT) have provided definitive evidence of causality in gut–mammary axis function, thereby substantiating the mechanistic relationships described above. Experiments demonstrating that transfer of dysbiotic gut microbiota from mastitis-affected animals to healthy recipients successfully induces mammary inflammation establish that gut microbiome composition directly influences mastitis susceptibility rather than merely reflecting disease-associated changes [76,78]. These causal relationships are further supported by complementary evidence examining the mammary microbiome itself. Interestingly, another study reported that changes in milk microbiota were significantly associated with mastitis [59], employing 16S rDNA high-throughput sequencing to perform correlation analysis of milk and gut microbial communities in mastitis-affected Holstein cows. This investigation revealed that alterations in the gut microbiome were paralleled by corresponding shifts in the mammary gland microbiota composition, with specific bacterial taxa showing concurrent enrichment or depletion across both anatomical sites [59].

These findings fundamentally validate the gut–mammary axis concept and provide strong rationale for therapeutic interventions targeting intestinal microbiome restoration as a strategy for mastitis prevention and management. In conclusion, the functional correlation between gut and milk microbiota establishes the intestinal microbiome as a critical determinant of mammary health, operating through integrated metabolic and immunological communication networks. Understanding these mechanistic connections provides an essential foundation for developing precision microbiota-based interventions that target the gut–mammary axis for mastitis prevention and treatment.

## 3. Enhancement of Microbiota and Their Association with Mastitis Management

The emerging understanding of the gut–mammary axis has fundamentally transformed our comprehension of mastitis pathogenesis, revealing intricate interconnections between gastrointestinal microbiota and mammary gland health that extend far beyond traditional infectious disease paradigms. Contemporary research demonstrates that mastitis development involves not only direct pathogenic microbial infections of mammary tissue but also encompasses sophisticated endogenous pathways mediated by rumen and gut microbiota dysbiosis, thereby establishing novel therapeutic targets for preventing and controlling this economically significant disease in dairy cattle operations [65]. This paradigm shift has opened new therapeutic avenues focused on restoring beneficial microbial communities and their protective metabolites rather than simply eliminating pathogenic organisms.

Restoration of beneficial microbiota represents a fundamental therapeutic strategy, with spore-forming bacteria transplantation demonstrating remarkable efficacy in reversing dysbiosis-induced mastitis susceptibility. Zhao C et al. [61] showed that spore-forming bacteria transplantation in dysbiotic mice restored gut microbial diversity, normalized DCA production and *TGR5* activation, while specific supplementation with *Clostridium scindens* increased mammary *TGR5* expression and DCA levels, reduced *S. aureus*-induced mammary damage, and restored tight junction proteins. This approach directly addresses the mechanistic deficits created by dysbiosis, particularly the disruption of secondary bile acid metabolism that normally provides anti-inflammatory protection to mammary tissue.

Understanding of harmful metabolic pathways has revealed specific targets for therapeutic intervention, particularly the role of succinate in exacerbating mastitis through novel microbiota-extracellular vesicle mechanisms. Qiu et al. [67] demonstrated that succinate, a metabolite elevated in SARA-associated mastitis, induces significant gut microbiota dysbiosis and exacerbates mammary inflammation through a microbiota-extracellular vesicle (mEV) mechanism. Succinate administration resulted in characteristic dysbiotic shifts featuring increased *Bacteroidetes* and decreased *Firmicutes* populations, while genus-level analysis revealed decreased beneficial commensals (*Lactobacillus* and *Phascolarctobacterium*) and increased potentially pathogenic taxa (norank_f_*Muribaculaceae* and *Prevotellaceae_UCG-001*). The dysbiotic microbiota produced increased quantities of mEVs containing lipopolysaccharides, which translocated across compromised intestinal barriers to mammary tissue and activated the TLR4/NF-κB inflammatory pathway.

### 3.1. Nutritional Interventions

Nutritional intervention strategies, including bioactive compounds, phytogenic substances, and probiotics, have demonstrated promising efficacy in mastitis management through modulation of immune function, metabolic processes, and microbiota composition [16,79,80]. Yeast fermentation products have emerged as effective interventions for correcting SARA-associated microbiota disruptions. Wu K. et al. [81] found that SARA decreased the abundance of *Firmicutes* and increased *Actinobacteriota* and *Bacteroidota* at the phylum level, with yeast fermentation product (YFF) supplementation effectively mitigating these detrimental changes by restoring microbial diversity and richness, increasing *Firmicutes* and *Actinobacteria* abundance, promoting beneficial genera like *Ruminococcus* and *Olsenella*, and suppressing SARA-associated genera. Specific bacterial genera correlated with inflammation, with genera like unclassified_o__*Oscillospirales* and *Denitrobacterium* showing negative correlation with inflammatory markers, while genera like *Lachnospiraceae_ND3007_group* and *Acetitomaculum* showed positive correlation, implicating them in mastitis promotion.

### 3.2. Probiotic and Prebiotic Interventions

Probiotics and prebiotics have emerged as promising therapeutic and preventive strategies for mastitis by modulating the mammary gland microbiome, enhancing immune function, and reducing pathogenic bacterial colonization [22,82,83,84]. Among these interventions, polysaccharide-based treatments represent a particularly promising therapeutic strategy, with multiple compounds demonstrating efficacy through gut microbiota modulation and short-chain fatty acid production. Li K et al. [85] showed that Astragalus polysaccharide (APS) effectively mitigated *S. aureus* infection in murine mammary glands through gut microbiota modulation, uniquely enriching *Ruminococcus bromii* in the gut and facilitating metabolism of SCFAs, particularly acetate and butyrate, which proved pivotal to APS’s protective effects. Similarly, Ran et al. [86] demonstrated that Angelica sinensis polysaccharide (ASP) effectively mitigated *S. aureus*-induced mastitis through targeted modulation of intestinal microbiota, with protective effects being entirely dependent on gut microbiota integrity, as evidenced by complete negation of benefits following antibiotic-induced microbiota disruption. ASP enhanced gut microbial diversity while promoting beneficial compositional shifts, specifically increasing *Lachnospiraceae_NK4A136* abundance and reducing *Erysipelatoclostridium* populations, with metabolomic analysis revealing elevated levels of bioactive metabolites including tabersonine and riboflavin.

Probiotic interventions have shown compelling therapeutic results through direct introduction of beneficial bacterial species and their bioactive products. Zhang B et al. [87] demonstrated that *Akkermansia muciniphila* and its derived outer membrane vesicles (AOMVs) showed significant potential, with Spearman’s correlation analysis revealing significant negative correlation between the relative abundances of *Verrucomicrobia* and *Akkermansia* and inflammatory cytokine levels in mastitis milk. Live *A. muciniphila* exhibited stronger anti-inflammatory effects compared to pasteurized preparations, notably inhibiting TLR4 and NF-κB signaling pathways, while administration significantly reduced somatic cell counts in mastitis-affected dairy cows, with four of five treated animals showing decreased counts below clinical thresholds and negative California Mastitis Test results. Marine-derived *Bacillus amyloliquefaciens*-9 (GB-9) demonstrated similar therapeutic potential in Saanen dairy goats, significantly reducing somatic cell counts and inflammatory markers including malondialdehyde, immunoglobulins (IgA, IgM), and pro-inflammatory cytokines (IL-2, IL-4, IL-6), while amplicon sequencing revealed alterations in fecal bacterial communities with *Bacteroides* and *Phascolarctobacterium* as primary responding genera [75].

Prebiotic supplementation has emerged as highly effective, with inulin demonstrating dose-dependent benefits through comprehensive modulation of both rumen and hindgut microbiota. Wang Y et al. [88] determined optimal therapeutic effects at 300 g/day, resulting in increased milk yield, protein, and lactose content alongside reduced somatic cell counts and decreased pro-inflammatory cytokines while enhancing anti-inflammatory IL-4 and antioxidant activity. Mechanistically, inulin enhanced rumen fermentation and promoted beneficial bacteria including *Prevotella*, *Butyrivibrio*, *Muribaculaceae*, and *Bifidobacterium* while suppressing pro-inflammatory taxa such as *Clostridia* UCG-014, *Streptococcus*, and *Escherichia-Shigella*. Wang Y et al. [89] further demonstrated that dietary inulin supplementation resulted in substantial alterations to milk microbiota composition, significantly reducing mastitis-causing and pro-inflammatory microbes including *Escherichia-Shigella*, *Pseudomonas*, *Rhodococcus*, and *Burkholderia-Caballeronia-Paraburkholderia*, while simultaneously increasing beneficial probiotics such as *Lactobacillus* and *Bifidobacterium*. Wang, Y. et al. [90] complemented these findings by investigating hindgut microbiome responses, showing increased abundance of beneficial *Bacteroides* and *Bifidobacteria* while decreasing potentially harmful taxa including *Paeniclostridium*, *Ruminococcaceae*, *Coprococcus*, and *Clostridia*, with elevated concentrations of beneficial short-chain fatty acids and reduced pro-inflammatory lipid oxidation products.

The mechanistic basis for prebiotic efficacy has been definitively established through microbiota transplantation studies. Zhao, C. et al. [66] showed that fiber-enriched high-inulin diet significantly alleviated *S. aureus*-induced mastitis in mice through gut microbiota-dependent mechanisms, as protective effects were completely abolished when gut microbiota was depleted with antibiotics, and fecal microbiota transplantation from high-inulin-treated mice successfully transferred protection to recipient animals. The protective mechanism involved increased production of short-chain fatty acids, particularly butyrate, which enhanced antimicrobial programs in macrophages by inhibiting histone deacetylase 3, while specific beneficial bacterial genera including *Prevotellaceae*, *Bacteroides*, and *Lachnospiraceae* were enriched and negatively correlated with mammary bacterial load and inflammation.

### 3.3. Plant-Derived Bioactive Compounds

**Figure 3:**
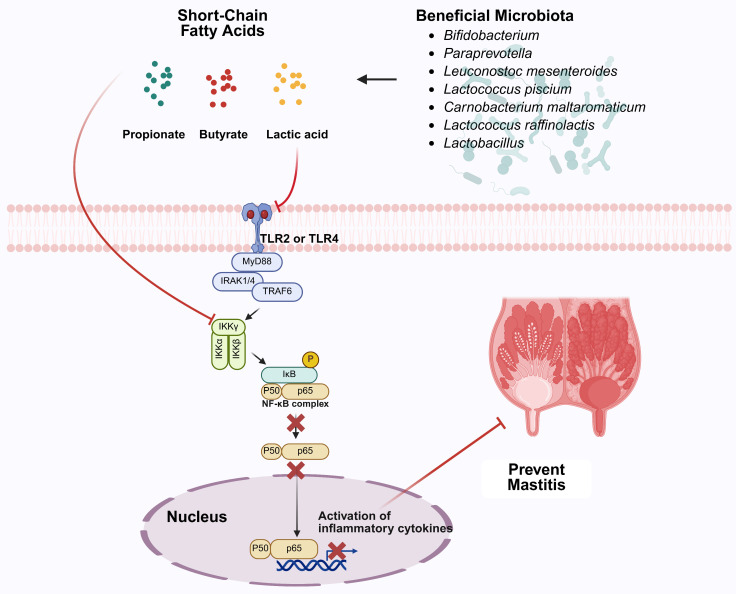
Short-chain fatty acids from beneficial microbiota modulate inflammatory pathways to prevent mastitis. This diagram illustrates how beneficial microbiota species (*Bifidobacterium*, *Parasutterella*, *Lactobacillus acidophilus*, *Lactococcus piscium*, *Garcionitricum multicarbonum*, *Lactococcus raffinolactis*, and *Lactobacillus* spp.) produce short-chain fatty acids (propionate-blue dots, butyrate-red dots, lactic acid-yellow dots) that modulate immune signaling pathways. These SCFAs interact with toll-like receptors (TLR2/TLR4) at the cell membrane (pink dotted line), triggering cytoplasmic signaling cascades involving MyD88, TRIF, and other intermediates that regulate nuclear transcription factors (NF-κB, IRF) to suppress inflammatory cytokine activation, ultimately preventing mastitis in mammary tissue.

Plant-derived bioactive compounds have demonstrated significant therapeutic potential through multiple interconnected mechanisms involving microbiota modulation, anti-inflammatory signaling, and barrier function restoration. Xie et al. [91] showed that hordenine, a phenylethylamine alkaloid naturally extracted from malt, effectively attenuated LPS-induced mammary tissue damage and restored blood–milk barrier integrity by inhibiting TLR4-MAPK/NF-κB signaling while simultaneously activating beneficial AMPK/Nrf2/HO-1 signaling pathways (Figure 3). Critically, hordenine modulated intestinal microbiota composition to increase beneficial *Lactobacillus* populations while reducing harmful inflammatory bacteria from *Desulfovibrionaceae* and *Enterobacteriaceae* families, leading to restoration of short-chain fatty acid levels, with butyrate demonstrating direct enhancement of tight junction protein expression. Similarly, Gao et al. [92] demonstrated that Forsythiaside A (FTA), a phenylethanol glycoside extracted from Forsythia, effectively mitigated LPS-induced mastitis through multiple interconnected protective mechanisms, significantly reducing inflammation and apoptosis levels, modulating PI3K/AKT/mTOR signaling pathways, and enhancing antioxidant capacity. FTA restored gut microbiota diversity by increasing beneficial bacteria such as *Lactobacillus* and *Bifidobacterium* while reducing pro-inflammatory taxa including *Helicobacteraceae* and *Desulfovibrionaceae*, normalizing the Firmicutes/Bacteroidetes ratio and elevating butyrate-producing genera like *Turicibacter*.

Additional plant compounds have shown similar efficacy through targeted microbiota modulation. Li K et al. [93] demonstrated that maslinic acid, an olive-derived compound, significantly altered gut microbiota structure, specifically increasing the abundance of potentially beneficial *Enterobacteriaceae* while decreasing harmful *Streptococcaceae*. This modulation towards a more favorable microbial profile coincided with reduced mammary inflammation and protection of the blood–milk barrier, alongside direct inhibition of mammary inflammatory pathways including *NLRP3*, AKT/NF-κB, and MAPK. Ran et al. [94] showed that hesperetin treatment restored healthy gut microbiota by reversing α-diversity loss, restructuring the microbial community, specifically suppressing harmful *Enterobacteriaceae* while enriching beneficial taxa like *Lactobacillaceae* (including *Lactobacillus johnsonii*) and *Lachnospiraceae* (SCFA-producers), with microbiota rebalancing correlating with inhibition of mammary TLR4/NF-κB inflammatory signaling pathway and preservation of tight-junction proteins crucial for blood–milk barrier integrity (Figure 3). Yu et al. [95] demonstrated that dihydromyricetin (DMY), a flavonoid monomeric compound, effectively ameliorated subclinical mastitis in dairy cows through complementary antioxidant and microbiota-modulating mechanisms. DMY supplementation significantly reduced milk somatic cell counts while enhancing serum antioxidant capacity by increasing total antioxidant capacity (T-AOC) and catalase (CAT) activities and reducing malondialdehyde (MDA) levels. The compound modulated gut microbiota composition by promoting beneficial genera including *Coprococcus* and *Roseburia* while reducing potentially harmful *Cyanobacteria*, *Proteobacteria*, and *Dehalobacterium* abundance. Metabolomic analysis revealed that DMY intervention decreased pro-inflammatory metabolites including arachidonic acid analogs and ω-6 polyunsaturated fatty acids while increasing anti-inflammatory compounds such as secondary bile acids and antioxidant vitamins, demonstrating its therapeutic potential as a dietary supplement for managing mammary inflammation through enhanced antioxidant activity and improved gut microbial community structure [95].

Traditional herbal compounds have provided additional therapeutic mechanisms. Sun et al. [96] demonstrated that *Abrus cantoniensis* total flavonoids (ATF) effectively reduced mammary tissue damage and inhibited secretion of inflammatory cytokines including TNF-α, IL-1β, and IL-6 in LPS-induced mastitis models, suppressing the CD14/TLR4/NF-κB/MAPK signaling pathway while enhancing tight junction protein expression in the blood–milk barrier, with intestinal microbiota analysis revealing regulatory effects on microbial composition. Xiang Y et al. [97] showed that *Pulsatilla chinensis* extract (PCE) demonstrated alternative treatment potential for S. aureus-induced mastitis through gut microbiota modulation, with principal coordinate analysis revealing that PCE treatment maintained intestinal microbiota structures closely resembling healthy controls, while mastitis model groups exhibited significant structural deviations. Mastitis development was characterized by significant increases in potentially pathogenic populations including *Proteobacteria*, *Deferribacterota*, and notably *Planctomycetota*, which appeared exclusively in diseased states, with comprehensive correlation analysis demonstrating that *Actinobacteriota* and *Bacteroidota* showed significant negative correlations with inflammatory markers, while *Firmicutes*, *Fusobacteriota*, and *Proteobacteria* demonstrated significant positive correlations with disease parameters.

Building on these findings, research into traditional Chinese herbal formulations has revealed multi-target therapeutic mechanisms that offer comprehensive treatment strategies [98]. Zhao G et al. [99] demonstrated that Gongying San (GYS) significantly increased milk yield, lactose, and milk protein while decreasing somatic cell counts, decreasing serum levels of lipopolysaccharide, interleukins (IL-2, IL-4, IL-8, IL-10), tumor necrosis factor-α, and malondialdehyde while increasing superoxide dismutase concentration. Microbiota analysis showed increases in *UCG-010* and *Blautia* and decreases in *Bacteroides*, *Lachnospiraceae*, and *Agathobacter*, with fecal untargeted metabolomics revealing that GYS supplementation primarily downregulated inflammation-related metabolism, including arachidonic acid and choline metabolism.

Direct intramammary interventions have also shown promise through local microbiome modulation. Zhang H et al. [100] reported that matrine-chitosan hydrogels demonstrated efficacy in modulating local microbiomes in dairy cows with subclinical mastitis, achieving significant reductions in somatic cell counts and microbial diversity, with treatment decreasing pathogenic microorganisms (*Aerococcus*, *Corynebacterium_1*, *Staphylococcus*, *Firmicutes*) while increasing beneficial *Proteobacteria*. Metabolomic analysis identified 74 differentially expressed metabolites, including upregulated sphingolipids, glycerophospholipids, flavonoids, and fatty acyls associated with glycerophospholipid and sphingolipid metabolic pathways, consistent with matrine’s antimicrobial and anti-inflammatory properties. The effects of various therapeutic interventions—namely nutritional interventions, probiotic and prebiotic interventions, plant-derived bioactive compounds, and direct intramammary interventions—on microbiota composition and subsequent mastitis development are presented in Table 2.

## 4. Conclusions

Mastitis extends beyond localized mammary infection, involving systemic microbiome disruption across mammary and intestinal sites, characterized by altered *Firmicutes*-to-*Proteobacteria* ratios and depletion of beneficial taxa (*Lactobacillus*, *Bifidobacterium*). Fecal microbiota transplantation experiments confirm that dysbiotic gut communities directly induce mammary inflammation, demonstrating the gut–mammary axis’s critical role.

This axis operates through metabolite signaling (SCFAs, bile acids), immune cell trafficking, bacterial translocation, and neuroendocrine coordination. Therapeutic interventions show significant efficacy: dietary fiber, probiotics (*Lactobacillus*, *Bifidobacterium*, *Bacillus* species), prebiotics, and plant-derived bioactives restore microbiome homeostasis while providing antimicrobial and immunomodulatory effects.

This paradigm shift reframes mastitis as a systems-level dysbiosis disorder rather than a simple infection. Future management strategies emphasize microbiome optimization and colonization resistance over antimicrobial pathogen elimination, offering sustainable alternatives that address antimicrobial resistance while promoting animal health and productivity.

## Figures and Tables

**Table 1:** Microbiota differences/associations with mastitis.

Animal Species	Sample Type	Health Status Comparison	Key Microbiota Differences/Associations	Reference
Cows/Mice	Gut	Mastitis vs. Healthy	↑ *Escherichia_Shigella*, ↓ *Roseburia; R. intestinalis* protective via butyrate production	[23]
Dairy cows	Milk	SCC levels (<100 K vs. 100–200 K vs. >200 K)	SCC < 100 K: ↑ *Bifidobacterium, Lachnospiraceae_AC2044*; Higher SCC: increased inflammatory gene expression	[24]
Yaks	Milk	Healthy vs. Subclinical vs. Clinical mastitis	Healthy: *Firmicutes* (39.7%), *Proteobacteria* (60.17%); Mastitis: *Proteobacteria* ↑ 89.32% (subclinical), 95.36% (clinical); *Firmicutes* ↓ 10.49%, 2.92%	[28]
Sahiwal cattle	Milk	Healthy vs. Subclinical vs. Clinical mastitis	Healthy: *Proteobacteria* (56.48%), *Firmicutes* (15.87%); Clinical: *Proteobacteria* (2.68%), *Firmicutes* (64%); Subclinical: intermediate patterns	[29]
Nili Ravi buffalo	Milk	Healthy vs. Subclinical vs. Clinical mastitis	Healthy: *Proteobacteria* dominant, *Streptococcus* (11.60%); Clinical: ↑ *Streptococcus* (33.96%), *Staphylococcus, Corynebacterium*	[30]
Holstein cows	Milk and Feces	Healthy vs. mastitis	Milk: ↑ *Firmicutes*, ↑ *Cyanobacteria*, ↑ *Streptococcus*, ↓ *Macrococcus caseolyticus.* Feces: No significant differences	[31]
Multiple breeds	Milk	Healthy vs. Subclinical mastitis	↑ *Firmicutes* in subclinical; ↑ *Proteobacteria* in healthy; 45 taxonomic biomarkers identified	[32]
Dairy cows	Milk	Non-subclinical vs. Subclinical mastitis	Non-subclinical: *Anthropi* spp., *P. azotoformans, P. fragi* dominant; Subclinical: *P. azotoformans, Mycobacterium bovis, P. koreensis*	[33]
Dairy cows	Milk	Healthy vs. Subclinical mastitis	↑ *Corynebacterium bovis, C. xerosis* (10-fold), *Streptococcus dysgalactiae, S. uberis;* Core mastitis microbiota: *Lactobacillus acidipiscis, Staphylococcus hominis*	[34]
Dairy cows	Milk	Healthy vs. GBS subclinical mastitis	↓ *Proteobacteria, Actinobacteria, Acidobacteria;* ↑ *Firmicutes, Streptococcus*, trends toward ↑ *Turicibacter, Enterococcus*	[35]
Dairy cows	Milk	Healthy vs. Subclinical vs. Clinical mastitis	↑ *Hymenobacter, Lachnospiraceae NK4A136* in mastitis; ↓ *Ralstonia, Lachnospiraceae NK3A20, Acetitomaculum, Massilia, Atopostipes*	[36]
Dairy cows	Milk and Teat canal	Healthy vs. Clinical mastitis	Inverse relationship: ↓ milk diversity = ↑ inflammation; *Sphingobacterium* negatively associated with diversity	[37]
Dairy cows	Milk	Healthy vs. Mastitis	Mastitis: *S. aureus, Aerococcus* spp., *Streptococcus* spp.; Surprising *S. thermophilus* in high SCC	[38]
Dairy cows	Milk	Clinical mastitis polymicrobial	486 cultures, 11 genera; 63.6% biofilm-forming; *S. aureus* most prolific biofilm former (18.8%)	[39]
Goats	Milk	Healthy vs. Subclinical vs. Clinical vs. Gangrenous	*Staphylococcus* dominant all groups; ↑ *Mycoplasma* in clinical; Novel *Escherichia/Shigella-Enterococcus* association in gangrenous	[40]
Dairy cows	Milk	Healthy vs. Mastitis across lactation	Mastitis: fewer bacterial taxa, ↓ diversity throughout lactation, *Proteobacteria* (9.1–95.4% vs. 24.0–92.9% healthy), *Firmicutes* (1.4–50.7% vs. 3.1–35.9% healthy)	[41]
Dairy cows	Milk	Clinical mastitis vs. Healthy	Mastitis: 363 vs. 146 strains in healthy; 68% unreported/opportunistic strains; 14 archaeal, 14 viral genera unique	[42]
Dairy cows	Milk	SCC levels (<2 × 10^5^ vs. >2 × 10^5^ vs. >5 × 10^5^)	SCC <2 × 10^5^: *Actinobacteriota* dominant; SCC 2 − 5 × 10^5^: *Firmicutes* dominant; SCC > 5 × 10^5^: *Firmicutes = Proteobacteria*	[43]
Holstein cows	Milk	High vs. Low resilience to mastitis	Low resilience: ↑ *Mycoplana, Rhodococcus;* High efficiency: ↑ *Aerococcus, Corynebacterium, Facklamia, Psychrobacter*	[45]
Humans	Breast tissue	Granulomatous vs. Acute mastitis vs. Controls	*Corynebacterium* >1% in 34.1% GM patients, *C. kroppenstedtii* predominant species	[46]
Dairy cows	Gut	Healthy vs. Subclinical mastitis	↑ *Cyanobacteria, Proteobacteria, Succinivibrio, Lactobacillus_iners;* ↓ *Paraprevotella*, *Coprococcus*, *Succiniclasticum*, *Desulfovibrio*, and *Bifidobacterium_pseudolongum*	[47]
Dairy cows	Rumen	Low vs. High SCC	↑ *Bacteroidetes, Firmicutes, Lachnospiraceae, Prevotella, Rumiclostridium* in H-SCC group	[48]
Dairy cows	Rumen	Subclinical vs. Clinical mastitis	Clinical: ↑ *Lachnospiraceae, Moraxella, Neisseriaceae*; ↓ beneficial SCFA producers	[49]
Cows/Mice	Rumen/Gut	SARA-mastitis vs. Healthy	↑ *Moraxellaceae*, ↓ *Prevotellaceae*; Sialic acid promotes ↑ *Enterobacteriaceae, Akkermansiaceae*	[50]
Holstein cows	Rumen/Feces/Milk	SARA vs. Controls	↑ *Stenotrophomonas* in rumen; Barrier disruption allowing bacterial translocation	[51]
Mice	Gut	Vagotomy-induced mastitis	↓ *Firmicutes, Proteobacteria;* ↑ *Campylobacterota*, Rikenellaceae_RC9_gut_group	[52]
Dairy cows	Rumen	Healthy vs. Subclinical vs. Clinical mastitis	Clinical: ↑ *Lachnospiraceae, Moraxella, Neisseriaceae*; ↓ *Prevoterotoella_1, Bifidobacterium*	[53]
Mice	Gut	S. aureus mastitis vs. Controls	↑ *Enterobacter*, ↓ short-chain fatty acids (SCFA)-producing bacteria (*Firmicutes, Bacteroidetes*)	[54]
Mice	Gut and Mammary gland	Antibiotic-treated vs. Controls	↓ *Firmicutes*, ↓ *Lactobacillaceae;* ↑ *Proteobacteria, Bacteroidota, Campylobacterota*	[55]
Dairy cows	Rumen and Feces	Healthy vs. Mastitis	Rumen: *Moryella* characteristic in mastitis; Feces: *Aeriscardovia*, *Lactococcus, Bacillus* in healthy	[56]
Rats	Gut	Healthy vs. Mastitis	↑ *Proteobacteria* phylum triggering metabolic disruptions	[57]
Dairy cows	Milk and Feces	Healthy vs. Subclinical vs. Clinical mastitis	Milk: ↑ *Proteobacteria*, ↓ *Firmicutes*, *Actinobacteriota, Bifidobacterium* in mastitis; Feces: ↑ UCG-010, *Bacteroides, Prevotella* in clinical mastitis	[58]
Dairy cows	Milk and Gut	Healthy vs. Mastitis	Milk: ↑ *Sphingomonas*, *Stenotrophomonas*; Feces: ↑ *Alistipes, Flavonifractor, Agathobacter, Pygmaiobacter*	[59]
Buffalo	Gut	Healthy vs. Mastitis	↑ *Muribaculaceae, Eubacterium_nodatum, Lachnoclostridium_10, Pichia*; ↓ *Ruminococcus_2, Candidatus_Stoquefichus, Turicibacter*	[60]
Cows/Mice	Multiple	Clinical mastitis vs. Healthy	Cows: *P. aeruginosa, L. crispatus, K. oxytoca*; Mice: *Muribaculum, Duncaniella, B. animalis, E. coli, S. aureus*	[62]
Cows/Mice	Rumen	Clinical mastitis vs. Healthy	↑ *Proteobacteria* in rumen; Dysbiosis-derived LPS promotes mastitis via TLR4-cGAS-STING-NF-κB/NLRP3	[63]
Humans/Mice	Gut	Mastitis vs. Controls	↑ *Firmicutes*/*Bacteroidetes* ratio, ↑ *Actinobacteria*, ↓ *Verrucomicrobia*, ↓ *Ruminococcus, Faecalibacterium;* ↑ *Parabacteroides*	[64]
Dairy cows and mice	Feces	Mastitis infected cow’s feces transplantation to mice	Identified key bacterial genera (*Chrysobacterium, Christensenellaceae_R-7_group, Prevotella*) as biomarkers. Revealed endogenous pathway mediated by rumen microbiota dysbiosis in mastitis development. Transplantation from mastitis cows induced mammary inflammation in mice.	[65]

“↓” shows decrease, “↑” indicate increase.

**Table 2:** Effects of various treatments on microbiota and mastitis management.

Treatment	Animal Model	Key Findings Associated with Mastitis Treatment	Reference
Fiber-enriched diet	Mice	✧ Prevent *Staphylococcus aureus*-induced mastitis ✧ Enriched *Prevotellaceae*, *Bacteroides*, and *Lachnospiraceae.* ✧ Increased butyrate production, enhanced antimicrobial programs in macrophages. ✧ Effects abolished with antibiotic-induced microbiota depletion.	[66]
Secondary Bile Acids/Clostridium scindens	Mice	✧ Restored secondary bile acid-producing bacteria. ✧ Increased mammary TGR5 expression and DCA levels. Activated TGR5-cAMP-PKA signaling, inhibited NF-κB/NLRP3. Restored tight junction proteins.	[67]
Bacillus amyloliquefaciens-9 (GB-9)	Saanen dairy goats	✧ Enhanced Bacteroides and Phascolarctobacterium populations. ✧ Reduced SCC, inflammatory markers (IL-2, IL-4, IL-6). Decreased immunoglobulins (IgA, IgM) and malondialdehyde. ✧ Effects mediated through Bacteroides-dependent immune modulation.	[75]
Yeast fermentation product (YFF)	Dairy goats	✧ Restored microbial diversity, increased *Firmicutes* and *Actinobacteria*. ✧ Promoted beneficial genera (*Ruminococcus, Olsenella*), suppressed SARA-associated genera. Reduced LPS levels, inflammatory cytokines, and oxidative stress.	[81]
Astragalus Polysaccharide (APS)	Mice	✧ Enriched *Ruminococcus bromii*, facilitated SCFA metabolism (acetate and butyrate). Reduced S. aureus infection, suppressed inflammation, restored blood–milk barrier integrity. ✧ Protective effects dependent on gut microbiota.	[85]
Angelica sinensis Polysaccharide (ASP)	Mice	✧ Increased Lachnospiraceae_NK4A136, reduced Erysipelatoclostridium. ✧ Enhanced gut microbial diversity. ✧ Elevated bioactive metabolites (tabersonine, riboflavin). ✧ Effects entirely dependent on gut microbiota integrity.	[86]
*Akkermansia muciniphila*	Dairy cows and mice	✧ Enhanced *Verrucomicrobia* abundance, negatively correlated with inflammatory cytokines. ✧ Inhibited TLR4 and NF-κB pathways. ✧ Reduced SCC ✧ Effects mediated through outer membrane vesicles (AOMVs).	[87]
Inulin (Rumen Microbiome)	Dairy cows	✧ Promoted propionate/butyrate-producing species (*Prevotella, Butyrivibrio*). ✧ Enhanced commensal bacteria (*Muribaculaceae, Bifidobacterium*). ✧ Suppressed pro-inflammatory taxa (*Clostridia UCG-014, Streptococcus*). ✧ Increased milk yield and quality.	[88]
Inulin Supplementation	Dairy cows	✧ Reduced mastitis-causing microbes (*Escherichia-Shigella, Pseudomonas*). ✧ Increased beneficial probiotics (*Lactobacillus, Bifidobacterium*). ✧ Enhanced SCFA production, reduced inflammatory mediators.	[89,90]
Hordenine	Mice	✧ Increased beneficial *Lactobacillus*, reduced Desulfovibrionaceae and Enterobacteriaceae. ✧ Restored SCFA levels (butyrate, acetate, propionate). ✧ Inhibited TLR4-MAPK/NF-κB, activated AMPK/Nrf2/HO-1 pathways. ✧ Enhanced tight junction proteins.	[91]
Forsythiaside A (FTA)	Mice	✧ Increased Lactobacillus and Bifidobacterium, reduced Helicobacteraceae and Desulfovibrionaceae. ✧ Normalized Firmicutes/Bacteroidetes ratio. Enhanced SCFA levels, reduced circulating LPS. ✧ Modulated PI3K/AKT/mTOR pathways.	[92]
Maslinic acid	Mice	✧ Increased beneficial *Enterobacteriaceae*, decreased harmful bacteria like *Streptococcaceae*. ✧ Altered gut microbiota structure (β-diversity changes). ✧ Reduced mammary inflammation (IL-6, IL-1β, TNF-α). Inhibited NLRP3, AKT/NF-κB, MAPK pathways.	[93]
Hesperetin	Mice	✧ Restored α-diversity, enriched *Lactobacillaceae* and *Lachnospiraceae*. ✧ Suppressed harmful *Enterobacteriaceae*. ✧ Inhibited TLR4/NF-κB pathway. Preserved tight-junction proteins (occludin, claudin-3, ZO-1).	[94]
*Abrus cantoniensis* total flavonoids (ATF)	Mice	✧ Increased beneficial Lactobacillus and norank_f_Muribaculaceae. ✧ Reduced harmful taxa (Lachnospiraceae, Bacteroides, Helicobacter). ✧ Suppressed CD14/TLR4/NF-κB/MAPK pathway. Enhanced tight junction proteins.	[95]
*Pulsatilla chinensis* extract (PCE)	Mice	✧ Maintained healthy microbiota structure, prevented pathological shifts. ✧ Reduced *Proteobacteria*, *Deferribacterota*, and *Planctomycetota.* ✧ Enhanced anti-inflammatory microbiota communities. ✧ Dual mechanism: pathogen inhibition + microbiota preservation.	[97]
Gongying San (GYS)	Holstein cows	✧ Increased UCG-010 and *Blautia*, decreased *Bacteroides* and *Lachnospiraceae.* ✧ Reduced SCC, LPS, inflammatory cytokines (IL-2, IL-4, IL-8, TNF-α). ✧ Enhanced milk yield, lactose, and protein. Downregulated arachidonic acid metabolism.	[99]
Matrine-chitosan Hydrogels	Dairy cows	✧ Decreased pathogenic microorganisms (Aerococcus, Corynebacterium_1, Staphylococcus). ✧ Increased beneficial Proteobacteria. ✧ Reduced SCC and microbial diversity. ✧ Enhanced sphingolipids and glycerophospholipids.	[100]

## Data Availability

No new data were created or analyzed in this study. Data sharing is not applicable to this article.
